# Shared genetic architecture between Alzheimer’s disease and psychiatric disorders revealed by multi-trait genome-wide analyses

**DOI:** 10.1186/s12920-026-02354-1

**Published:** 2026-04-28

**Authors:** Huajun Zhang, Liangzhuo Xie, Facai Meng, Jiangli Cui, Bo Ma, Yu Wang, Kai Zhang, Xingyu Miao

**Affiliations:** 1 Neurosurgery Department, Shaanxi Provincial People’s Hospital, Xi’an, Shaanxi China; 2 Longhua Hospital Shanghai University of Traditional Chinese Medicine, Shanghai, China

**Keywords:** Alzheimer’s disease, Psychiatric disorders, Genetic correlation, Colocalization analysis, Expression quantitative trait loci (eQTL)

## Abstract

**Objective:**

Clarify the shared genetic architecture between Alzheimer’s disease (AD) and major psychiatric disorders.

**Methods:**

We integrated large GWAS summary datasets derived predominantly from individuals of European ancestry for AD and eight psychiatric disorders; estimated genome-wide genetic correlations with LDSC/HDL; mapped local genetic correlations with SUPERGNOVA; ran pairwise Multi-trait analysis of GWAS (MTAG (AD with ADHD, BIP, MDD, PTSD, SCZ)) followed by FUMA; performed cross-trait colocalization with HyPrColoc and trait–eQTL colocalization using GTEx v8 (49 tissues).

**Results:**

HDL identified significant genome-wide correlations for five AD–psychiatric pairs, led by AD–MDD; LDSC was significant for AD–MDD. We mapped 18 locally correlated regions. MTAG yielded 33 AD-associated loci (118 SNPs), including 12 novel (e.g., *RAB27B*/rs12968702, *PTCH1*/rs3824488, *EP300*/rs12157997), and 336 psychiatric-trait signals across 265 loci. HyPrColoc detected 74 AD–psychiatric colocalized regions (40 with PP ≥ 0.8), with 13 driven by a single candidate causal variant. Trait–eQTL colocalization prioritized 25 genes in 122 associations; brain-tissue signals implicated *P4HTM*, *GPX1*, *CCDC71*, and—at an AD–SCZ locus—*ADAM10*.

**Conclusion:**

AD shares substantial pleiotropic architecture with psychiatric disorders, particularly MDD. Integrative multi-trait association, colocalization, and tissue-specific eQTL evidence highlights convergent mechanisms—exosome biology (*RAB27B*), astrocytic Ca²⁺ signaling (*P4HTM*), and non-amyloidogenic APP processing (*ADAM10*)—and nominates testable therapeutic targets.

**Supplementary Information:**

The online version contains supplementary material available at 10.1186/s12920-026-02354-1.

## Introduction

Alzheimer’s disease (AD) is the most common cause of dementia, accounting for an estimated 60–80% of diagnoses and affecting ~ 50 million people worldwide [1]. As a prototypical neurodegenerative disease, AD is pathologically characterized by extracellular β amyloid (Aβ) deposition and neurofibrillary tangles composed of hyperphosphorylated tau [2]. Epidemiologically, multiple studies have linked a range of psychiatric disorders to increased risk of AD [3, 4]. Clinically, anxiety prevalence in AD rises from about 9% in the preclinical phase to almost 40% in the mild-to-severe phases [5]; Moreover, AD patients with comorbid clinical depression have been observed to carry a higher Aβ plaque burden [6]. Mechanistically, chronic stress promotes phosphorylation of tau and amyloid precursor protein (APP), induces synaptic dysfunction, and may accelerate disease progression [7]. Given the extensive interdigitation of emotional and cognitive networks, AD manifestations extend beyond cognitive decline to include a spectrum of neuropsychiatric symptoms (NPS) [8]. Collectively, epidemiologic evidence, clinical features, and biological plausibility support a tight, multilayered relationship between AD and psychiatric symptoms. Elucidating the mechanisms underlying AD–psychiatric comorbidity is therefore of clear importance.

At the genetic level, both AD and several psychiatric disorders are highly heritable [9–11]. Genome-wide association studies (GWAS) indicate not only robust disorder-specific risk variation but also nontrivial genetic overlap and shared determinants. A conditional/conjunctional false discovery rate (FDR) analysis identified 16 shared SNPs between AD and schizophrenia (SCZ), with approximately half showing concordant risk directions, suggesting shared molecular mechanisms [12]. Using conditional FDR, two additional shared loci were discovered between AD and bipolar disorder (BIP), implicating *MARK2* and *VAC14* [13]. More recently, local genetic correlation analyses (LAVA) revealed significant shared signals between AD and SCZ across multiple genomic regions [14]. Additionally, recent studies have further documented genetic correlations and specific shared loci between AD and other psychiatric conditions, such as major depressive disorder [15, 16]. Nevertheless, the specific regulatory mechanisms and molecular processes linking AD with psychiatric disorders remain unclear. Integrative analyses of their shared genetic architecture can help disentangle common versus disorder-specific etiologies and, by highlighting functionally relevant shared targets and pathways, may ultimately enable interventions that address both AD and psychiatric disorders.

In this study, we assembled the largest publicly available GWAS summary datasets for AD and eight psychiatric disorders—attention deficit hyperactivity disorder (ADHD), anorexia nervosa (AN), autism spectrum disorder (ASD), schizophrenia (SCZ), major depressive disorder (MDD), bipolar disorder (BIP), post-traumatic stress disorder (PTSD), and tourette syndrome (TS)—and conducted a systematic, multi-phenotype investigation. Using a multi-trait GWAS framework in concert with complementary statistical-genetic approaches, we interrogated genome-wide global and local architectures at the SNP and gene levels with the aim of extending current knowledge by identifying novel pleiotropic variants and loci, and by prioritizing candidate pleiotropic genes and their putative tissues of action through an integrated analytical pipeline.

## Methods

### GWAS data and quality control

**Fig. 1:**
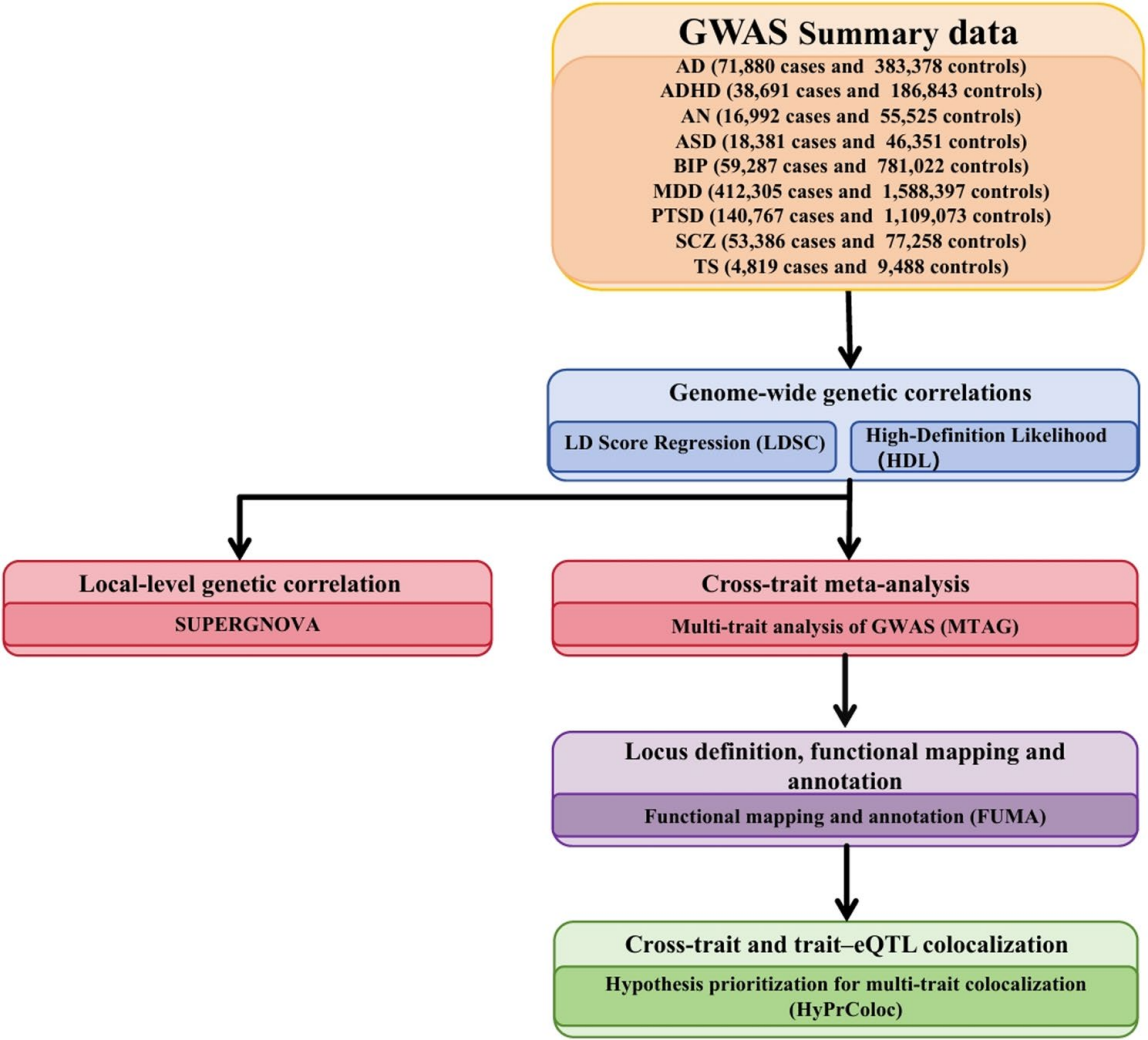
Schematic overview of the multi-stage genetic analysis pipeline

An overview of the overall analytical workflow is presented in Fig. 1. We extracted GWAS summary statistics from publicly available sources. All summary statistics used in this study were derived from cohorts with predominantly European ancestry participants. Ethical approval had been obtained in the original studies. Psychiatric disorder data were taken from the latest releases of the Psychiatric Genomics Consortium (PGC) [17], including SCZ [18], ADHD [19], AN [20], ASD [21], BIP [22], MDD [23], PTSD [24], and TS [25]. All PGC summary statistics were downloaded from the consortium’s official repository for researchers: https://pgc.unc.edu/for-researchers/download-results/. GWAS data for AD were downloaded from https://ctg.cncr.nl, comprising 71,880 AD cases and 383,378 controls [26]. Detailed descriptions of all GWAS summary datasets are provided in Supplementary Table 1. The sample sizes for each trait are summarized in Table 1. Briefly, the GWAS datasets comprised 71,880 AD cases and 383,378 controls (Jansen et al., 2019), 38,691 ADHD cases and 186,843 controls, 59,287 BIP cases and 781,022 controls, 412,305 MDD cases and 1,588,397 controls, 140,767 PTSD cases and 1,109,073 controls, 53,386 SCZ cases and 77,258 controls, 16,992 AN cases and 55,525 controls, 18,381 ASD cases and 46,351 controls, and 4,819 TS cases and 9,488 controls.

**Table 1:** Genetic correlation estimated by HDL and LDSC from GWAS summary datasets of Alzheimer’s disease and psychiatric disorders

Diseases	Abbreviations	PMID	N_cases	N_control	Notes
Attention deficit hyperactivity disorder	ADHD	36,702,997	38,691	186,843	
Anorexia nervosa	AN	31,308,545	16,992	55,525	
Autism spectrum disorder	ASD	30,804,558	18,381	46,351	
Bipolar disorder	BIP	39,843,750	59,287	781,022	
Major depressive disorder	MDD	39,814,019	412,305	1,588,397	
Posttraumatic stress disorder	PTSD	38,637,617	140,767	1,109,073	
Schizophrenia	SCZ	35,396,580	53,386	77,258	
Tourette syndrome	TS	30,818,990	4819	9488	
Alzheimer’s disease	AD	30,617,256	71,880	383,378	
Attention deficit hyperactivity disorder	ADHD	36,702,997	38,691	186,843	

For each analysis, we retained only SNPs present in both the AD dataset and the psychiatric dataset under consideration. To avoid confounding from the complex linkage disequilibrium (LD) structure of the major histocompatibility complex (MHC) region (chromosome 6: 25–34 Mb), SNPs in this region were excluded from all analyses. We also excluded rare variants (minor allele frequency, MAF < 0.01) as well as insertions and deletions (indels).

### Genome-wide genetic correlations

To assess genetic correlations between AD and the eight psychiatric disorders, we applied cross-trait LD Score Regression (LDSC) [25]. Using LD scores for HapMap3 SNPs estimated in European-ancestry individuals from the 1000 Genomes Project [27], LDSC regresses the product of Z-scores from two traits on the LD score to estimate the genetic covariance; the genetic correlation is then obtained by standardizing this covariance by the square root of the product of the traits’ SNP heritabilities [28].

We further validated genome-wide correlations using High-Definition Likelihood (HDL) [28], which, unlike LDSC, uses the full LD matrix rather than LD scores alone and jointly estimates SNP heritability and genetic correlation under a multivariate normal model via maximum likelihood. Compared with LDSC, HDL reduces the variance of genetic correlation estimates by approximately 60%—roughly equivalent to a 2.5-fold increase in effective sample size—thereby providing greater statistical power [28].

For both LDSC and HDL, we applied Bonferroni correction and set the significance threshold at *P* < 6.25 × 10⁻³ (0.05/8) to control the type I error rate across eight trait pairs. Trait pairs with P-values below this threshold in either HDL or LDSC were considered significant and were carried forward to downstream analyses.

### Local-level genetic correlation analysis

Genome-wide correlation estimates reflect average effects across the genome and may miss local, heterogeneous pleiotropy. Therefore, for trait pairs showing significant global genetic correlation, we performed local analyses using SUPERGNOVA [29] across 2,353 approximately independent LD blocks [30]. SUPERGNOVA accounts for LD by decorrelating local Z-scores with eigenvectors of the local LD matrix and then estimating local genetic covariance via weighted least-squares regression, from which local genetic correlation is derived [31]. After Bonferroni correction, the significance threshold was *P* < 2.12 × 10⁻⁵ (0.05/2353).

### Cross-trait meta-analysis

We conducted five pairwise MTAG analyses [30] between AD and each of the five psychiatric disorders that showed significant genome-wide correlations. MTAG jointly analyzes multiple traits using only GWAS summary statistics, thereby increasing statistical power. Built-in bivariate LDSC regression is used to adjust for potential sample overlap and reduce bias from shared participants or structural confounding. Each MTAG run produced trait-specific summary statistics (SNP effect estimates), which were used in subsequent analyses.

### Functional mapping and annotation using FUMA

We used FUMA (Functional Mapping and Annotation) on all MTAG-derived summary statistics to identify independent lead SNPs, perform functional annotation, and conduct gene mapping [32]. From the GWAS results, independent significant SNPs were defined as those with *P* < 5 × 10⁻⁸ and pairwise LD r² < 0.6. Among these, SNPs with pairwise r² < 0.1 were designated lead SNPs. SNPs in LD (r² > 0.6) with any independent significant SNP (including those present in the 1000 Genomes reference panel) were assigned to the same LD block. Two LD blocks were merged into a single genomic locus if the distance between them was ≤ 500 kb. Pairwise LD analyses used the 1000 Genomes European reference data [27]. Within each LD block, the lead SNP with the smallest P-value was defined as the top signal, and remaining lead SNPs were considered secondary signals. Gene mapping was performed by aligning SNP chromosomal positions to the reference genome.

To ensure robustness, we retained an MTAG-identified locus only if the corresponding SNP also showed association in the original single-trait GWAS (*P* < 0.01) with a consistent effect direction. For AD, we aggregated independent signals across all pairwise MTAG analyses and merged lead-SNP loci into a single genomic locus when inter-locus distances were < 500 kb.

### Novel loci definition and replication

A locus was considered novel if (i) its top signal reached genome-wide significance in MTAG (*P* < 5 × 10⁻⁸), (ii) the corresponding variant showed nominal significance (*P* < 0.01) in the original single-trait GWAS with the same effect direction, and (iii) it was outside ± 1 Mb of any previously reported locus. Signals within ± 1 Mb of known loci were not counted as novel.

### Cross-trait and trait–eQTL colocalization

We performed colocalization using HyPrColoc (Hypothesis Prioritization for multi-trait Colocalization) [33], a deterministic Bayesian framework that leverages multi-trait GWAS summary statistics to efficiently identify clusters of traits sharing a causal variant within a genomic region. We centered analyses on each MTAG top signal, testing a ± 200 kb window for cross-trait colocalization. Default settings were used, including variant-specific priors that assume the probability of a single variant colocalizing across multiple traits decreases as the number of traits increases. Two key priors were specified: the prior probability that a variant is associated with any one trait (*P* = 1 × 10⁻⁴) and the conditional prior that it is associated with an additional trait given association with one (Pᶜ = 0.02).

We considered posterior probability (PP) > 0.5 as evidence of reliable colocalization at a locus, and categorized evidence as moderate (0.5 < PP < 0.8) or strong (PP ≥ 0.8). A variant explaining ≥ 80% of the shared association signal was designated the candidate causal variant.

Building on trait–trait colocalization results, we further performed trait–eQTL colocalization by integrating AD and psychiatric traits with eQTL data from 49 GTEx v8 tissues [34]. Parameter settings mirrored those used above. This analysis aimed to identify potentially shared genes between traits and to characterize their tissue-specific expression profiles.

## Results

### Genetic correlation between AD and psychiatric disorders

Using publicly available GWAS summary statistics, we evaluated genetic correlations between AD and eight psychiatric disorders. At the genome-wide level, five trait pairs remained significant after Bonferroni correction. Specifically, HDL identified five significant pairs: AD–ADHD (*P* = 1.53 × 10⁻⁴), AD–BIP (*P* = 5.83 × 10⁻³), AD–MDD (*P* = 3.64 × 10⁻⁶), AD–PTSD (*P* = 3.11 × 10⁻⁶), and AD–SCZ (*P* = 1.86 × 10⁻³). LDSC reached correction-level significance only for AD–MDD (*P* = 3.10 × 10⁻³). Although the remaining four pairs did not survive multiple testing under LDSC, their P-values were all < 0.05 and the effect directions were broadly consistent with HDL, suggesting concordant trends and robustness of the findings (Table 1).

At the local level, we identified 18 associated regions across the five significant trait pairs. One region was detected for AD–ADHD on chr11 (p14.2–p14.1). Fourteen regions were identified for AD–MDD on chr1 (p34.2), chr3 (p21.2–p21.1), chr4 (q31.23–q31.3), chr4 (p16.3), chr6 (q16.1), chr6 (p24.2–p24.1), chr7 (q22.2–q22.3), chr9 (p21.3), chr11 (q24.2), chr14 (q32.31–q32.33), chr14 (q13.3–q21.1), chr15 (q21.2), chr21 (q22.2), and chr22 (q12.3–q13.1). Two regions were found for AD–PTSD on chr1 (q24.2) and chr10 (q25.3–q26.11), and two for AD–SCZ on chr10 (p12.1) and chr16 (p11.2) (Supplementary Data 2).

### Cross-trait joint analysis identifies novel loci for AD and multiple psychiatric disorders

**Fig. 2:**
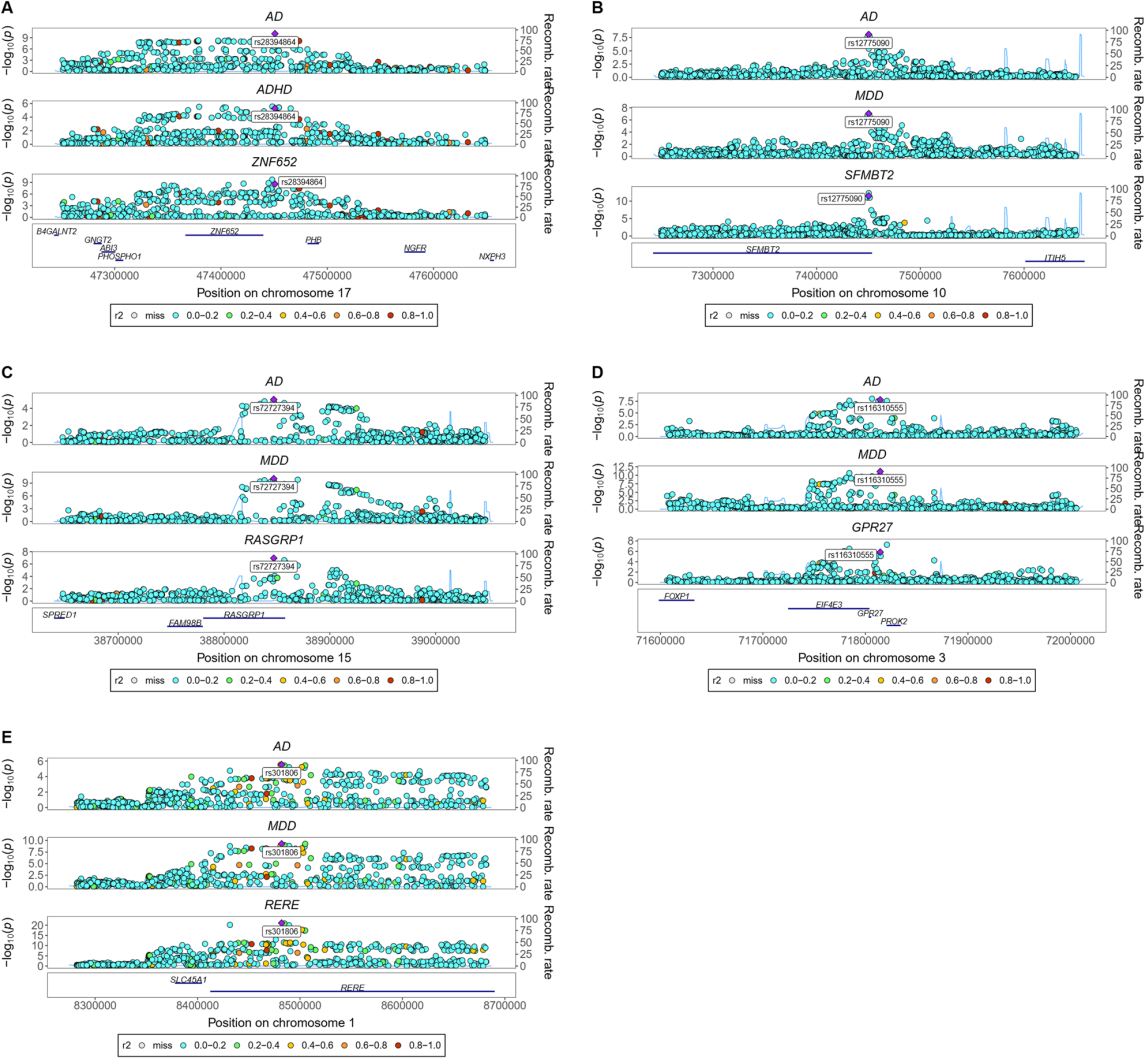
The five dominant causal variant loci with concentrated posterior weights—rs28394864 (located in *ZNF652*), rs116310555 (located in *GPR27*), rs72727394 (located in *RASGRP1*), rs12775090 (located in *SFMBT2*), and rs301806 (located in *RERE*)—are illustrated in the regional plot. This figure shows the colocalized loci of Alzheimer’s disease (top), psychiatric disorders (middle), and expression quantitative trait loci (eQTLs, bottom) in relevant tissues. The x-axis represents the chromosomal positions of single nucleotide polymorphisms (SNPs), and the y-axis represents the negative base-10 logarithm of the P value (–log10P) Figure 2 Regional plots of five colocalized loci shared between Alzheimer's disease (AD) and psychiatric disorders. For each locus, the top panel shows AD association signals (--log₁₀P), the middle panel shows the corresponding psychiatric disorder association signals, and the bottom panel shows expression quantitative trait locus (eQTL) signals in relevant tissues. The x‑axis represents chromosomal position, and the y‑axis represents --log₁₀P. (A) AD and ADHD colocalization at the ZNF652 locus (lead SNP rs28394864). (B) AD and MDD colocalization at the SFMBT2 locus (lead SNP rs12775090). (C) AD and MDD colocalization at the RASGRP1 locus (lead SNP rs72727394). (D) AD and MDD colocalization at the GPR27 locus (lead SNP rs116310555). (E) AD and MDD colocalization at the RERE locus (lead SNP rs301806)

We performed five pairwise multi-trait GWAS MTAG analyses between AD and ADHD, BIP, MDD, PTSD, and SCZ. MTAG results were visualized alongside the corresponding original GWAS findings (Fig. 2). Across all pairwise MTAG analyses, we identified 33 unique genetic loci associated with AD, corresponding to 118 distinct SNPs. Among these 33 AD loci, 12 were novel. Notably, within chr19: 45,020,859–45,734,751, a dense cluster of lead SNPs was observed, suggesting that this region is highly relevant to AD–psychiatric overlap (Supplementary Data 3). Among the novel loci, rs12968702 (*RAB27B*) showed the strongest association (Table 2).

**Table 2:** Novel genetic loci reaching genome-wide significance for association with the examined traits

SNP	Chr	Pos	EA	OA	Beta	*P*	Gene
Alzheimer’s disease							
rs13383529	2	22,650,358	T	C	0.009	1.38E-08	*AC068490.2*:*AC096570.2*
rs13399760	2	124,960,421	G	A	-0.011	1.27E-08	*CNTNAP5*
rs112669807	3	71,806,435	T	C	0.016	9.27E-09	*GPR27*
rs359539	3	155,282,432	A	G	-0.013	3.90E-09	*PLCH1*
rs7712317	5	153,246,661	C	T	-0.012	5.01E-09	*AC091962.3*
rs3824488	9	98,236,664	T	C	0.015	2.92E-08	*PTCH1*
rs3814877	16	30,042,677	T	G	-0.013	1.94E-09	*FAM57B*
rs35283920	16	70,687,195	T	C	0.012	2.21E-08	*IL34*
rs4311	17	61,560,763	T	C	-0.012	7.77E-09	*ACE*
rs12968702	18	52,487,062	C	T	-0.013	4.73E-10	*RAB27B*
rs62206419	20	36,235,683	T	C	0.010	3.07E-08	*GLRXP*
rs12157997	22	41,479,672	A	G	0.013	5.20E-09	*EP300*
Schizophrenia							
rs1901512	5	101,723,875	T	C	0.027	1.41E-09	*SLCO6A1*
rs500102	9	77,358,745	T	C	0.023	1.16E-08	*TRPM6*
Bipolar disorder							
rs10917509	1	19,992,066	T	C	0.010	2.91E-09	*HTR6*
rs11167957	5	146,436,523	C	T	-0.010	9.62E-10	*PPP2R2B*
rs898471	8	27,589,315	T	C	0.015	3.32E-08	*CCDC25*
rs4595478	10	111,800,145	T	C	0.014	1.09E-10	*ADD3*
rs9896486	17	1,252,843	T	G	0.009	3.77E-08	*YWHAE*
Posttraumatic stress disorder							
rs116286607	2	60,177,131	A	G	0.010	1.91E-08	*RP11-444A22.1*
rs74446154	16	49,624,547	A	G	0.020	2.40E-08	*ZNF423*
Major depressive disorder							
rs28680958	1	173,848,808	A	G	-0.008	5.34E-10	*ZBTB37*
rs2963662	5	175,850,820	T	C	-0.008	2.40E-09	*CLTB*
rs6930109	6	103,030,195	A	G	-0.006	2.48E-09	*RP11-793L10.1*
rs7461469	8	3,328,265	T	C	0.007	3.44E-08	*CSMD1*
rs7147873	14	47,275,276	T	C	0.006	1.62E-08	*MDGA2*
SNP	Chr	Pos	EA	OA	Beta	P	Gene
Alzheimer’s disease							
rs13383529	2	22,650,358	T	C	0.009	1.38E-08	*AC068490.2*:*AC096570.2*
rs13399760	2	124,960,421	G	A	-0.011	1.27E-08	*CNTNAP5*
rs112669807	3	71,806,435	T	C	0.016	9.27E-09	*GPR27*
rs359539	3	155,282,432	A	G	-0.013	3.90E-09	PLCH1
rs7712317	5	153,246,661	C	T	-0.012	5.01E-09	*AC091962.3*
rs3824488	9	98,236,664	T	C	0.015	2.92E-08	*PTCH1*
rs3814877	16	30,042,677	T	G	-0.013	1.94E-09	*FAM57B*
rs35283920	16	70,687,195	T	C	0.012	2.21E-08	*IL34*
rs4311	17	61,560,763	T	C	-0.012	7.77E-09	*ACE*
rs12968702	18	52,487,062	C	T	-0.013	4.73E-10	*RAB27B*
rs62206419	20	36,235,683	T	C	0.010	3.07E-08	*GLRXP*
rs12157997	22	41,479,672	A	G	0.013	5.20E-09	*EP300*
Schizophrenia							
rs1901512	5	101,723,875	T	C	0.027	1.41E-09	*SLCO6A1*
rs500102	9	77,358,745	T	C	0.023	1.16E-08	*TRPM6*
Bipolar disorder							
rs10917509	1	19,992,066	T	C	0.010	2.91E-09	*HTR6*
rs11167957	5	146,436,523	C	T	-0.010	9.62E-10	*PPP2R2B*
rs898471	8	27,589,315	T	C	0.015	3.32E-08	*CCDC25*
rs4595478	10	111,800,145	T	C	0.014	1.09E-10	*ADD3*
rs9896486	17	1,252,843	T	G	0.009	3.77E-08	*YWHAE*
Posttraumatic stress disorder							
rs116286607	2	60,177,131	A	G	0.010	1.91E-08	*RP11-444A22.1*
rs74446154	16	49,624,547	A	G	0.020	2.40E-08	*ZNF423*
Major depressive disorder							
rs28680958	1	173,848,808	A	G	-0.008	5.34E-10	*ZBTB37*
rs2963662	5	175,850,820	T	C	-0.008	2.40E-09	*CLTB*
rs6930109	6	103,030,195	A	G	-0.006	2.48E-09	*RP11-793L10.1*
rs7461469	8	3,328,265	T	C	0.007	3.44E-08	*CSMD1*
rs7147873	14	47,275,276	T	C	0.006	1.62E-08	*MDGA2*

In addition, we detected 336 signals associated with various psychiatric disorder traits across 265 genetic loci (Supplementary Data 4), including 2 novel loci for SCZ, 5 for BIP, 2 for PTSD, and 5 for MDD (Table 2). Among these, rs1901512 (*SLCO6A1*), rs4595478 (*ADD3*), rs116286607 (*RP11-444A22.1*), and rs28680958 (*ZBTB37*) exhibited the strongest trait-specific associations. Overall, at the genome-wide level, nine distinct AD SNPs (across nine loci) were associated with at least one of the tested psychiatric disorder traits (Supplementary Data 5).

### Colocalization analysis identifies shared genetic loci between AD and psychiatric disorders

Applying HyPrColoc to 33 AD loci and 265 psychiatric-trait MTAG-associated loci, we identified 74 colocalized regions between AD and psychiatric traits (posterior probability, PP > 0.5; Supplementary Data 6). The majority of signals occurred between AD and MDD (*n* = 58). Across all loci, 40 showed strong colocalization evidence (PP ≥ 0.8). Among novel loci, the region at chr9: 98,236,664 ± 200 kb (*PTCH1*; lead SNP rs3824488) showed significant AD–MDD colocalization (PP = 0.89), and chr1: 173,848,808 ± 200 kb (*ZBTB37*; lead SNP rs28680958) likewise displayed strong AD–MDD colocalization (PP = 0.81).

Among these 40 loci with strong evidence, 13 were driven by a single candidate causal variant explaining more than 80% of the shared signal (%PP > 0.8; Supplementary Data 6), including: rs324017 (*NAB2*; AD–SCZ; PP = 0.98, %PP = 1), rs76400970 (*RP11-702F3.4*; AD–MDD; PP = 0.97, %PP = 1), rs11257952 (*CAMK1D*; AD–MDD; PP = 0.99, %PP = 0.99), rs13056300 (*Y_RNA*; AD–MDD; PP = 0.92, %PP = 0.99), rs12157997 (*EP300*; AD–MDD; PP = 0.92, %PP = 0.99), rs12144378 (*SMYD2*; AD–MDD; PP = 0.96, %PP = 0.98), rs28394864 (RP11-81K2.1; AD–ADHD; PP = 0.81, %PP = 0.95), rs11599236 (*SORCS3*; AD–MDD; PP = 0.93, %PP = 0.91), rs11877758 (*CELF4*; AD–MDD; PP = 0.80, %PP = 0.89), rs4948485 (*ARID5B*; AD–MDD; PP = 0.96, %PP = 0.87), rs1373285 (*AL450423.1*; AD–MDD; PP = 0.97, %PP = 0.84), rs12775090 (*SFMBT2*; AD–MDD; PP = 0.99, %PP = 0.82), and rs28680958 (*ZBTB37*; AD–MDD; PP = 0.81, %PP = 0.81) ( Supplementary Data 6).

### Gene-expression colocalization prioritizes shared causal genes between AD and psychiatric traits

To identify candidate pleiotropic causal genes at colocalized loci, we performed trait–eQTL colocalization using GTEx v8 eQTLs across 49 tissues, focusing on genes proximal to the 74 AD–psychiatric colocalized regions for four disorders (ADHD, MDD, PTSD, SCZ). We found 18 loci in which AD and at least one psychiatric trait colocalized with the expression of one or more genes in the same tissue, yielding 122 associations involving 25 genes. Stratified by disorder: ADHD 2 (2 loci, 2 genes), MDD 68 (12 loci, 17 genes), PTSD 11 (1 locus, 1 gene), and SCZ 41 (3 loci, 6 genes) (Supplementary Data 7).

Notable examples include rs116310555, which supported AD–MDD colocalization with *GPR27* across multiple tissues; rs641325, which supported AD–PTSD colocalization with *MED19*; and rs9420, which supported AD–MDD colocalization with *MED19* in multiple tissues. These findings suggest that the implicated loci may contribute to shared AD–psychiatric risk by modulating gene expression in specific tissues (Supplementary Data 7).

We also observed five loci with colocalization driven by a single, high-posterior-probability SNP (i.e., the same SNP jointly explained the GWAS and eQTL signals with most posterior weight concentrated on that variant): rs28394864–*ZNF652*, rs116310555–*GPR27*, rs72727394–*RASGRP1*, rs12775090–*SFMBT2*, and rs301806–*RERE*.

Focusing on brain tissues, we identified 20 associations. Of particular interest, rs7617480 colocalized with *P4HTM* (PP = 0.85) and *GPX1* (PP = 0.55) in brain cerebellum, with *CCDC71* in brain putamen (PP = 0.76), and with *P4HTM* in brain cerebellar hemisphere (PP = 0.56). Another locus, rs6131010, colocalized with *CD40* in brain cerebellum (PP = 0.72). For rs442495, we observed colocalization with *ADAM10* in brain spinal cord (PP = 0.64), and with *MINDY2* in brain hippocampus (PP = 0.52) and brain caudate (PP = 0.72).

## Discussion

In this study, we conducted a large-scale cross-trait genetic analysis. Overall, using LDSC and HDL, we identified significant genetic correlations for five trait pairs (AD–ADHD, AD–BIP, AD–MDD, AD–PTSD, and AD–SCZ) and, with SUPERGNOVA, further pinpointed 19 locally correlated regions across these pairs.

Subsequently, Applying MTAG and FUMA at the genome-wide significance threshold, we identified 12 previously unreported AD-associated loci. Among them, rs12968702 (*RAB27B*) showed the strongest association with AD and also reached genome-wide significance for MDD; regional cross-trait colocalization provided statistical support for a shared causal signal between the two traits. Prior work shows that exosomes originate from multivesicular bodies (MVBs) containing intraluminal vesicles; fusion of MVBs with the plasma membrane releases these vesicles as exosomes [35]. Inhibiting exosome biogenesis and secretion can markedly reduce the spread of tau pathology and the aggregation of amyloid-β in AD models [36]. *RAB27B*, a member of the Rab GTPase family, is critical for exosome secretion by promoting the peripheral positioning of MVBs and their docking at the plasma membrane, suggesting a potential role in AD pathogenesis [37]. Moreover, an independent causal-inference framework (Mendelian randomization, Bayesian colocalization, and Steiger filtering) has implicated *RAB27B* protein levels in brain tissue as causally related to MDD [38]. However, we currently lack tissue eQTL colocalization evidence directly linking rs12968702 to *RAB27B* expression; thus, whether this locus contributes to AD–MDD comorbidity via *RAB27B* requires further validation.

When systematically evaluating psychiatric disorders in relation to AD, we observed that MDD exhibited the richest set of shared pleiotropic signals with AD, highlighting the importance of MDD-related pathways in explaining AD comorbidity; this finding is consistent with gene-level analyses [39, 40]. Clinically, numerous studies and meta-analyses report that depression is associated with elevated risks of cognitive impairment, AD, and other dementias [41, 42]. Biologically, potential links between MDD and AD include cortisol dysregulation, neuroinflammation, and hypothalamic–pituitary–adrenal (HPA) axis abnormalities [43, 44]. In addition, the antidepressant citalopram has been shown to reduce Aβ production rates in human cerebrospinal fluid and to inhibit plaque growth in aged transgenic AD mice [45]. Nonetheless, existing evidence remains largely correlational and is insufficient to establish a unified pathophysiology or a definitive causal chain between the two disorders.

We found that rs7617480 colocalized with *P4HTM*, *GPX1*, and *CCDC71* in different brain regions with MDD and AD, suggesting gene–tissue–specific pleiotropy at this locus. Neural network dysfunction and altered functional connectivity are early features of AD, and correcting astrocytic Ca²⁺ signals can partially restore these abnormalities [46]. Notably, *P4HTM* is highly expressed in the brain and acts as a regulator of Ca²⁺ signaling in astrocytes [47], implicating it in early AD mechanisms. Multiple studies also link astrocytic Ca²⁺ signaling to depression [48–50]. It is therefore plausible that *P4HTM*, by modulating astrocytic Ca²⁺ dynamics, may influence both AD pathology and MDD development, offering a mechanistic clue to their comorbidity. *GPX1* (glutathione peroxidase 1), broadly localized to the cytosol and mitochondria, reduces intracellular ROS via antioxidant activity and may confer protection against AD; its enzymatic activity has also been associated with depressive symptoms [51–53]. Accordingly, *GPX1* may affect shared susceptibility to AD and MDD by maintaining brain-region-specific redox homeostasis. *CCDC71* (Coiled-Coil Domain Containing 71) belongs to the intracellular coiled-coil protein family [54]. Existing evidence suggests that *CCDC71* variation associates with depressive symptoms and that rs7617480 is a prioritized SNP in this region; however, no direct link with AD has been established [55].

Beyond AD–MDD, in the AD–SCZ analyses we observed that rs442495 colocalized with multiple genes (*ADAM10* and *MINDY2*) across brain regions. *ADAM10* is a principal neuronal α-secretase mediating the non-amyloidogenic pathway by cleaving within the Aβ sequence of *APP*, thereby preventing Aβ peptide formation and releasing neuroprotective s*APP*α [56]. *ADAM10* is considered a therapeutic target in AD because it initiates the non-amyloidogenic pathway and may prevent downstream disease processes [57]. The Notch pathway plays a key role in neurodevelopment and adult brain homeostasis and appears dysregulated in the peripheral blood of patients with SCZ [58]. Consistently, *ADAM10* is the rate-limiting S2 protease for Notch activation and is essential for maintaining neural stem cell pools during early embryonic cortical development, suggesting a potential shared role for *ADAM10* in both SCZ and AD [59]. By contrast, we found no supportive evidence for a disease link with *MINDY2*; although it is a K48-specific deubiquitinase with clear relevance to protein homeostasis [60], there is as yet no direct evidence connecting *MINDY2* to pathological changes in AD or SCZ.

In the AD–SCZ cross-trait analysis, we thus observed colocalization of rs442495 with *ADAM10* and *MINDY2* across different brain regions, indicating that a single variant may influence multiple molecular pathways via region/cell-type-specific transcriptional regulation. First, *ADAM10*, as the major α-secretase, drives the non-amyloidogenic processing of *APP*, blocking Aβ generation and releasing sAPPα [56]; accordingly, it is a therapeutic target that both initiates the non-amyloidogenic route and may interrupt downstream pathology [57]. Second, Notch signaling is vital for neurodevelopment and adult homeostasis and is dysregulated in SCZ peripheral blood [58]; *ADAM10*, as the rate-limiting S2 protease for Notch, is indispensable for maintaining neural stem cell pools during embryonic cortical development [59]. In contrast, *MINDY2*’s disease relevance remains unsubstantiated; despite its biologically meaningful role as a K48-specific DUB in protein quality control [60], no direct evidence presently links it to AD or SCZ pathology.

Taken together, we observed broad genome-wide genetic correlations between neuropsychiatric disorders and AD and, on this basis, further explored pleiotropic genetic signals and genes. LDSC and HDL indicated significant global correlations mainly between ADHD, BIP, MDD, PTSD and AD. SUPERGNOVA detected 19 significant locally correlated regions. MTAG–FUMA analyses identified 118 distinct SNPs mapping to 33 AD-specific loci (including 12 novel loci), and 336 distinct SNPs mapping to 265 psychiatric-specific loci (including 5 novel loci). Building on these results, colocalization analyses identified 74 shared AD–psychiatric regions, 40 of which had PP > 0.8. Subsequent trait–eQTL colocalization across 49 GTEx tissues yielded 122 associations involving 25 genes. This work has several limitations. First, our GWAS datasets were restricted to individuals of European ancestry, limiting generalizability to other populations. Second, there was substantial sample-size imbalance across the contributing summary statistics, which may reduce statistical efficiency. Third, our analyses considered only SNPs with MAF > 0.01 and excluded the MHC region, potentially missing rare variants and disease-specific loci that could deepen understanding of shared genetic architecture. Fourth, some reported associations have not yet been replicated in independent, large European-only meta-analyses. Finally, the biological functions of the pleiotropic genetic markers identified here remain to be elucidated in in vitro and in vivo experiments.

## Supplementary Information

Supplementary Material 1.

## Data Availability

Processed result tables generated in this study are available from the corresponding author on reasonable request.

## Abbreviations

Aβ: Amyloid—β
AD: Alzheimer’s disease
ADAM10: A Disintegrin and Metalloprotease 10
ADHD: Attention Deficit Hyperactivity Disorder
AN: Anorexia Nervosa
APP: Amyloid Precursor Protein
ASD: Autism Spectrum Disorder
BIP: Bipolar Disorder
eQTL: Expression Quantitative Trait Locus/Loci
EP300: E1A Binding Protein p300
FDR: False Discovery Rate
FUMA: Functional Mapping and Annotation (of genetic associations)
GPX1: Glutathione Peroxidase 1
GTEx: Genotype—Tissue Expression (project)
GWAS: Genome—Wide Association Study/Studies
HDL: High—Definition Likelihood (genetic correlation method)
HPA: Hypothalamic—Pituitary—Adrenal (axis)
HyPrColoc: Hypothesis Prioritization for multi—trait Colocalization
IL34: Interleukin—34
LDSC: Linkage Disequilibrium Score Regression
LD: Linkage Disequilibrium
MAF: Minor Allele Frequency
MDD: Major Depressive Disorder
MHC: Major Histocompatibility Complex
MINDY2: Motif Interacting with Ubiquitin—containing Novel DUB family, member 2
MTAG: Multi—Trait Analysis of GWAS
MVB: Multivesicular Body
NPS: Neuropsychiatric Symptoms
PGC: Psychiatric Genomics Consortium
PP: Posterior Probability
PTCH1: Patched 1
PTSD: Post—Traumatic Stress Disorder
RAB27B: RAB27B, member RAS oncogene family
SCZ: Schizophrenia
SFMBT2: Scm Like with Four MBT Domains 2
SNP: Single Nucleotide Polymorphism
SUPERGNOVA: (Tool for) local genetic correlation analysis
TS: Tourette Syndrome
